# *Murradambirra Dhangaang* (make food secure): Aboriginal community and stakeholder perspectives on food insecurity in urban and regional Australia

**DOI:** 10.1186/s12889-022-13202-z

**Published:** 2022-05-28

**Authors:** Simone Sherriff, Deanna Kalucy, Allison Tong, Nawazish Naqvi, Janice Nixon, Sandra Eades, Tangerene Ingram, Kym Slater, Michelle Dickson, Amanda Lee, Sumithra Muthayya

**Affiliations:** 1 The Sax Institute, Sydney, Australia; 2 Children’s Hospital Westmead Clinical School, Sydney Medical School, The University of Sydney, Sydney, Australia; 3 Sydney School of Public Health, Faculty of Medicine and Health, The University of Sydney, Sydney, Australia; 4 Department of Medicine, University of Toronto, Toronto, Canada; 5 Melbourne School of Population and Global Health, Melbourne University, Melbourne, Australia; 6 Riverina Medical and Dental Aboriginal Corporation, Wagga Wagga, Australia; 7 Tharawal Aboriginal Corporation, Airds, Australia; 8 School of Public Health, The University of Queensland, Brisbane, Australia

**Keywords:** Food insecurity, Diet inequality, Aboriginal and Torres Strait Islander, Indigenous, Stakeholders, Food systems, Chronic disease, Urban, Qualitative

## Abstract

**Background:**

It is widely acknowledged that the invasion by colonial powers of the Australian continent had profound and detrimental impacts on Aboriginal Communities, including food security. Policies of successive governments since European arrival have since further exacerbated the situation, with food insecurity now affecting 20–25% of Aboriginal and Torres Strait Islander people. Food insecurity contributes to long-term impacts on health, in particular diet-sensitive chronic diseases. This study aimed to describe Aboriginal community and stakeholder perspectives on food insecurity to get a better understanding of the key contributing factors and recommendations for potential strategies to address this issue in Aboriginal communities in urban and regional Australia.

**Methods:**

Semi-structured interviews were conducted with 44 participants who were purposively selected. This included Aboriginal people in two communities and both Aboriginal and non-Aboriginal stakeholders from local food relief agencies, food suppliers, schools, and government in an urban and regional location in NSW. A conceptual framework was developed from literature on food security, and sensitizing concepts of availability, affordability, accessibility and acceptability or the lack thereof of healthy food were used to elicit responses from the participants. Interview transcripts were analysed thematically.

**Results:**

All participants felt strongly that food insecurity was a major problem experienced in their local Aboriginal communities. Five core areas impacting on food security were identified: trapped in financial disadvantage; gaps in the local food system; limitations of non-Aboriginal food relief services; on-going impacts of colonization; and maintaining family, cultural and community commitments and responsibilities. Participants suggested a number of actions that could help ease food insecurity and emphasized that Aboriginal values and culture must be strongly embedded in potential programs.

**Conclusions:**

This study found Aboriginal families in urban and regional Australia are experiencing food insecurity on a regular basis, which is impacted by a range of socio-economic, environmental, systemic and cultural factors, as reported by the participants. Study findings highlight the need to address system level changes in the food environment and acknowledge Aboriginal history, culture and food preferences when considering the development of programs to alleviate food insecurity among Aboriginal people.

**Supplementary Information:**

The online version contains supplementary material available at 10.1186/s12889-022-13202-z.

## Background

Food insecurity is a serious challenge experienced by Aboriginal and Torres Strait Islander people amidst a wider growing crisis of hunger and food inequality in Australia [1]. As many as one in five Aboriginal and Torres Strait Islander people living in non-remote environments – who make up three-quarters of the total Aboriginal and Torres Strait Islander population in Australia –reported being food insecure in a national 2012–13 survey [1]. This compares to one in four among remote Aboriginal and Torres Strait Islander communities and one in twenty-five among the wider Australian population. Hunger and food insecurity has been on the rise worldwide over the past few decades, and the economic fallout and food crisis resulting from the COVID-19 pandemic has further exacerbated this problem globally and in Australia [2–4]. Food security exists “when all people, at all times, have physical and economic access to sufficient, safe and nutritious food to meet their dietary needs and food preferences for an active and healthy life” [5]. Four key components - food availability, accessibility, acceptability and adequacy are highlighted in this definition of food security by the Food and Agriculture Organisation of the United Nations.

Food insecurity can have significant lifelong consequences on the health and wellbeing of populations and, therefore, must be addressed as a significant public health priority by all levels of government. Mild to moderate food insecurity is associated with poor physical and mental health in both children and adults [6]. Children are particularly vulnerable to both the short and longer-term effects of food insecurity, resulting in anaemia, greater learning and behavioural problems, mental health issues and increased risk of hospitalization, [7–9]. Food insecurity is also directly related to higher rates of obesity and chronic disease in adults [6, 10]. Food-insecure households tend to respond by adjusting the food budget, decreasing dietary variety and increasing the consumption of cheap, energy dense foods [11]. National survey data show that about 95% of Aboriginal and Torres Strait Islander people (aged 15 years and over) report not eating adequate amounts of vegetables each day, with 41% of their energy intake derived from discretionary foods and drinks that tend to be high in added sugar, saturated fat, salt and/or alcohol [12], often as a result of unequal availability of, or access to, a healthy food supply [13, 14]. The inequality in diet and food insecurity experienced by Aboriginal and Torres Strait Islander people likely contributes to the differential impact on diet-sensitive cardiovascular diseases and other chronic disease, and related burden of disease [15, 16].

Aboriginal and Torres Strait Islander people, who before European invasion and colonization had managed and sustained their food environment to meet their needs for over 60,000 years through a deep understanding of the land, water and connection to their Country (traditional lands), are now among the most socially and economically disadvantaged communities in Australia, and experience challenges purchasing adequate quantities of food, as well as adequate high quality healthy food due to financial and other constraints [17–20]. Food environments and food insecurity experienced by Aboriginal and Torres Strait Islander people in non-remote Australia also differ from those experienced in remote areas [21, 22]. While the urban food environment may offer a tremendous diversity of food for consumers, there are huge geographical disparities in access to fresh, healthy food, determined by the socioeconomic status (SES) of each region [23]. A study in an urban area of Australia found that people living in low-SES areas purchased a smaller variety of fruits and grocery foods that were low in fibre and high in fat, salt and sugar, compared to people in advantaged areas [24]. In that study, SES was associated with food purchasing behaviours, suggesting that a decrease in food availability, accessibility, and affordability, made the purchase of some types of foods more difficult in disadvantaged areas. Greater exposure to unhealthy foods and pervasive marketing of these foods in low-SES areas add to the complex, urban obesogenic food environment [25–28].

There has been limited research examining the key drivers of food insecurity among Aboriginal and Torres Strait Islander people living in non-remote areas and it is, therefore, important to gain a complete understanding of the issue, including underlying systemic and other factors, prior to attempting to develop and implement solutions [29]. Developing this requires a recognition that food security is very much a complex and dynamic issue at the intersection between social and cultural, economic, agricultural, nutrition and health sectors [20, 30]. Combining holistic Indigenous knowledge and addressing systemic factors across these multiple sectors [31, 32] can complement this approach to understanding food insecurity. This study, on the key drivers of food insecurity and recommendations for potential solutions, conducted in urban and regional New South Wales (NSW) aimed to describe the perspectives, knowledge and beliefs of Aboriginal families, Aboriginal Elders and Aboriginal staff members linked to two NSW Aboriginal Community Controlled Health Services (ACCHSs) [33], and those of a range of stakeholders in government and other organizations linked to the local food system. This much needed data can provide a better awareness of how to support Aboriginal communities in non-remote areas experiencing food insecurity and guide the development of community-led programs to strengthen local food systems.

## Methods

This research used a qualitative approach and was informed by decolonising research methodology. Utilising these methods, which challenge Western colonial constructs, allowed the research team to give a platform for Aboriginal community members to safely share their stories, stay as close as possible to the voices of the participants and allow their perspectives to inform the findings. This approach ensured the Aboriginal members of the project team and the communities involved played an integral part in the research process, leading all aspects of it and ensuring that the lived experience and worldviews of Aboriginal peoples were captured, in accordance with the literature [34–36].

For this paper we respectfully use the term Aboriginal hereafter to refer to the traditional custodians of lands in NSW, in line with current recommendations [37].

### Research team

Aligning with decolonising methodology, the first author (SS) an Aboriginal woman, a researcher and mother who lives in one of the study communities, analysed the data and drafted the paper from an Indigenous standpoint [38]. The study was conceptualised by the senior author (SM) who has extensive experience in public health nutrition and has worked in Aboriginal health research for 10 years in collaboration with the two partner Aboriginal communities. The research team brings together Aboriginal lived experience (SS, JN, SE, TI, KS, MD), ACCHS expertise (SS, JN, TI, KS), nutrition research (SE, AL, SM) and qualitative expertise (SS, DK, AT, NN, JN, MD, SM).

### Setting

The research was conducted in two communities in NSW with a higher than average Aboriginal population: Campbelltown, an urban outer suburb of Greater Sydney with a population of 78,849 people (4.7% Aboriginal) [39]; and Wagga Wagga, a large regional area located in inland NSW approximately 5 h drive from Sydney which has a population of 62,385 people (5.6% Aboriginal) [39]. The Aboriginal communities represented in this study have a weekly household income that is $105 lower (Campbelltown) and $242 lower (Wagga Wagga) when compared to the median weekly household income for their respective Local Government Areas [40].

### Participant selection and recruitment

Ethics approval for this study was provided by the Aboriginal Health and Medical Research Council of NSW (1226/16).

This study is embedded within the Study of Environment on Aboriginal Resilience and Child Health (SEARCH) [41, 42]. SEARCH is a large cohort study that has been co-created with four ACCHSs in NSW to assess the health and wellbeing of 1669 Aboriginal children and their families living in urban communities in NSW to inform the delivery of relevant programs and services [43]. Food and nutrition insecurity were reported by parents/caregivers in SEARCH to be the third most significant factor affecting the health and wellbeing of their children, after “loving family relationships” and “culturally competent healthcare” [44].

Participants for this study were recruited through two of the partner ACCHSs in SEARCH: Tharawal Aboriginal Corporation located in Campbelltown, and Riverina Medical and Dental Aboriginal Corporation in Wagga Wagga. ACCHSs provide holistic and culturally relevant programs and services to their local communities, which focus not just on the Western biomedical view of health, but take into account their spiritual, emotional, psychological, and physical wellbeing [45]. ACCHSs are run by an elected group of local Aboriginal people who sit on the board of directors and are set up to circumvent barriers faced by Aboriginal people in accessing mainstream health services [46]. Therefore, recruiting participants for this study through ACCHSs added a level of trust to the research that would not have been possible without this connection.

We used purposive sampling to recruit a range of Aboriginal community members, including Aboriginal Elders and families, ACCHS board members and ACCHS staff, and Aboriginal and non-Aboriginal representatives from food relief agencies, schools, local government, council, Local Health Districts, Primary Health Networks, and food suppliers. ACCHS staff identified local Aboriginal community members and other stakeholders who could offer insights about food insecurity in the local Aboriginal community, and a list of potential participants was developed. Participants were recruited via telephone or in person by an ACCHS or SEARCH staff member and were advised that the study was being conducted to gather Aboriginal community and stakeholders’ perspectives on food insecurity faced by the local Aboriginal community.

### Data collection

An interview guide (Supplementary file 1) was developed by the research team using the definition of food security described above and available literature on food insecurity using the sensitizing concepts [47] of availability, affordability, accessibility and acceptability or the lack thereof of healthy food, to guide the interview questions and inform analyses [5, 48]. It was refined through discussions with senior Aboriginal researchers and community leaders who have expertise in Aboriginal health, public health nutrition, qualitative research and epidemiology. Questions focused on participants’ knowledge and beliefs around food insecurity, challenges, and barriers to food security for themselves and for the local Aboriginal community, factors that influence it, and ideas for actions to improve the local food system.

Semi-structured one-on-one interviews were conducted between September 2017 to August 2018. They were conducted either face-to-face at the local ACCHSs, at a location convenient for the participant, or over the telephone by four researchers (SS, DK, JN and SM), two of whom are Aboriginal researchers (SS and JN). In line with decolonising methodology, all interviews had an Aboriginal researcher (SS or JN) conducting the questioning together with another researcher (DK or SM) to ensure cultural safety and the accurate interpretation of participant responses. Participation was voluntary and written, informed consent was obtained prior to the interview being conducted. Recruitment ceased when data saturation was reached, where interviews were no longer giving additional information to develop new themes but instead were endorsing already identified themes [49]. All interviews were audio-recorded and transcribed verbatim, with participant permission.

### Data analysis

Two authors (SS and NN) independently read all transcripts and two additional authors (DK and SM) independently read half of the transcripts each, and all developed preliminary themes relating to food security. Thematic analysis was used drawing on principles of grounded theory to guide the process [50]. Four authors (SS, DK, NN and SM) coded the transcripts line by line to inductively identify emerging themes. The researchers (SS, DK, NN, JN and SM) met regularly to refine themes and sub-themes. Each theme was developed with data from the transcripts by SS, DK NN and SM and then further refined through analytical discussions with AT. HyperRESEARCH, version 4.0.1 (Research-ware Inc., Randolph, MA, USA) was used to code the data (SS). Findings are reported using Sandelowski’s qualitative description approach [51], whereby data are represented in a way that most stakeholders would agree is acceptable and stays as near to the data as possible by summarising it in participants’ everyday terms [51]. The COnsolidated criteria for Reporting Qualitative Research (COREQ) framework was used to report this study [52].

**Fig. 1:**
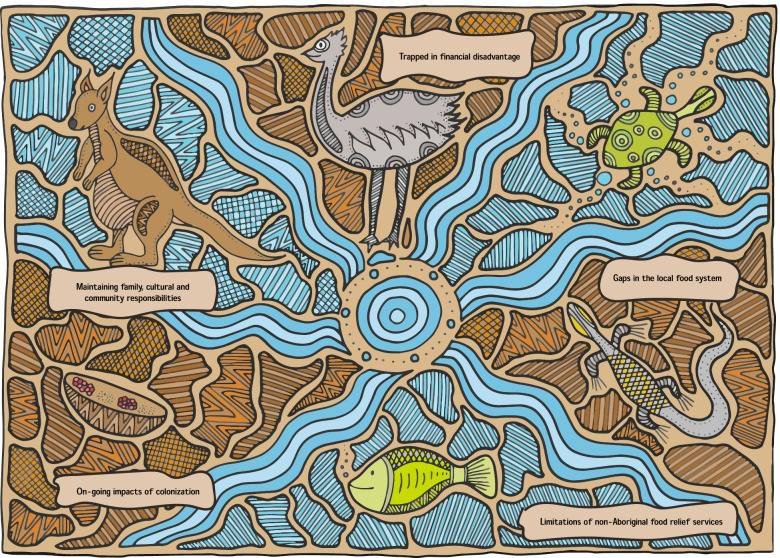
Key themes of food insecurity in two Aboriginal communities. Artwork by Shakara Montalto at Deadly Designs, a proud Gunditjmara woman**.** The artwork in Fig. 1 engages with a culturally grounded way of communication that uses an Aboriginal lens to help position the paper within an Aboriginal context. The browns and orange patterns represent the land, and the strong connection Aboriginal people have to Country (traditional lands), and the blue swirls and circles represent the rivers and water ways that sweep throughout Australia. The artwork depicts bush tucker (traditional, native foods) including the Kangaroo, Emu, Turtle, Goanna, Fish, and a Coolamon (curved piece of wood for collecting nuts, plants, seeds and carrying babies)

The decolonising methodological approach also guided the analysis process, with the research team discussing the initial findings with the partner ACCHSs during which the idea to use an artwork as a tool for dissemination and engagement was developed. Although this paper uses a Western written approach to tell a story of food security in two Aboriginal communities, Aboriginal people have used song, dance, and art to tell our stories for many generations. Therefore, the artwork below was created to support visual and verbal discussions with the communities around the research findings and is an example of our engagement with decolonising methodology. An artwork shown in Fig. 1 tells a story of food security experiences in the two Aboriginal communities and is used in the context of this study with permission from the artist.

## Results

Of the 52 people who were invited to participate, 44 (85%) agreed and completed the interview. The duration of interviews was on average 30 minutes. Participant characteristics are described in Table 1. Non-participation was due to scheduling conflicts, illness, and non-response.

**Table 1:** Participant characteristics (*n* = 44)

Characteristics	***n***	%
**Gender**		
Male	12	27%
Female	32	73%
**Aboriginal status**		
Aboriginal	22	50%
Non-Aboriginal	22	50%
**Age (years)**		
20–39	15	34%
40–49	10	23%
50–59	8	18%
60–69	6	14%
70+	5	11%
**Highest level of Education**		
Masters or above	6	14%
Bachelor’s degree	8	18%
Diploma	6	14%
Certificate/some College	5	11%
High school	15	34%
Primary school	1	2%
No answer	3	7%
**Stakeholder group**		
ACCHS staff and board members	9	20%
Aboriginal community members (Elders, parents, and caregivers)	13	30%
Food supplier, food relief organizations/charities	12	27%
Council, local government, LHD and Education sectors	10	23%

*ACCHS* Aboriginal Community Controlled Health Service, *LHD* Local Health District

All Aboriginal participants felt strongly that food insecurity was a major problem that many Aboriginal people in their communities were facing. Throughout the interviews, Aboriginal participants often recalled their own personal experiences with food insecurity and factors contributing to it and at other times they spoke about the experiences of others in their families or the wider Aboriginal community. A majority of the non-Aboriginal participants representing local stakeholders from council, government, the Department of Education, food suppliers, food relief organizations and charities spoke generally about the local food security system, rather than the specifics of food insecurity facing the local Aboriginal community. Therefore, most of our results and quotes are based on information shared by Aboriginal participants, demonstrating application of our decolonising methodology. A small number of non-Aboriginal participants had differing perceptions on the barriers to food security for Aboriginal families which have been described in the results.

We identified five core themes which were largely consistent across the two study sites: trapped in financial disadvantage; gaps in the local food system; limitations of non-Aboriginal food relief organizations; on-going impacts of colonization; and maintaining family, cultural and community commitments and responsibilities. The respective subthemes are included in the descriptions below*.*

### Theme 1: trapped in financial disadvantage

#### Caught in constant debt and struggling to survive

Participants felt families were running out of money before each pay cycle and were unable to purchase food, which was further intensified for single parent or single income families. They noted these financial struggles were due to high rates of unemployment and low income, often leading them to borrow money from family or friends and take short-term high interest loans to support their household. This made it more difficult for families to pay for necessities including food as families:

“ … *think that the best thing to do is go and get one of them daily loans to have money, then they're paying back triple the amount which leads them into debt. Then they can't buy anything … it's just a circle that just keeps going around and around”.* (Aboriginal community member)

#### Dominating and competing priorities draining the food budget

Participants explained that many Aboriginal families in their community struggled just to cover essential, fixed expenses (i.e. rent, childcare, other bills) and may prioritise financial resources towards fulfilling those essentials, which can deplete the food budget.

Participants described that some families worried about high utility costs associated with cooking food at home, and therefore chose quick cooking meal options that were often unhealthy.

Some participants felt that money used for tobacco, drugs, alcohol and gambling also put pressure on the family food budget and, was a major concern within their community:

*“ … it's hidden, un-talked about. I think gambling has the biggest impact within our Aboriginal [community], and it's not addressed”.* (Aboriginal LHD staff member)

Some participants spoke about how families felt pressure to keep up appearances with the latest trends in order to avoid judgement and do not want to be seen as ‘poor’ or ‘struggling’, which they felt resulted in some families purchasing items such as the latest televisions, gaming devices, mobile phones and iPads that put a strain on the budget. 

#### Rationing and relying on cheap, filling foods

Participants described rationing food to ensure there was enough to go around until more money or food could be sourced. This included parents/caregivers eating less or skipping meals for up to a few days at a time to ensure their children had enough food. Other strategies, such as attending playgroups and other community events that provide food, were used to ensure their children have a meal that day.

Participants explained that families felt resigned to choosing cheap, unhealthy foods that *“clog the kids up”* just to provide enough food for their families to survive and consequently this made them feel disheartened:

*“I had one mum who came in and said to me, I have five kids and yes I know I don't eat properly but if I buy myself a large hot chips and a loaf of white bread, my kids are all full and can go to bed happy and satisfied. You're telling me to eat more fresh fruit and vegetables I don't even know if they're going to eat it. I can't afford to buy it. I don't even know if I'm going to waste my money on it”.* (ACCHS staff member)

Participants felt families in their communities were generally big meat eaters but noted that good cuts of meat are often expensive and unaffordable for many family budgets. This leaves many to purchase cheap meat packs predominately filled with sausages, mince and cheaper processed meats:

*“If you've got eight people in your family, you're not going to go and buy eight T-bone steaks. You're going to buy a bucket load of rissoles or sausages to feed that family. I think that's what it comes down to”.* (Aboriginal community member)

### Theme 2: gaps in the local food system

#### Lack of healthy options and demotivation stemming from insufficient investment

Participants described the unavailability of healthy food options both in terms of takeaway shops and supermarkets in the suburbs where most Aboriginal families live. Participants felt that having only smaller independent supermarkets nearby meant that healthy food was often limited, stale, and expensive. As an Aboriginal Elder explained *“well you go to the supermarket, and it’s disgraceful. There’s nothing there. There’s nothing fresh. It’s mostly all tinned stuff”*. It was noted that while healthy food was more regularly available at larger supermarkets in neighbouring suburbs, it was still unaffordable for many families:

“ … *no one can afford to buy it, because the healthy food is so expensive. It's a big factor, like everyone wants all these kids to eat healthy, but they're not looking at the price of things”. (*Aboriginal community member*)*

Participants also described how many local shops had closed in the suburbs where most Aboriginal families live and that there had been a lack of ongoing commitment from their local councils to ensure adequate infrastructure in their communities. As an ACCHS staff member explained, “*lots of shops have closed in our suburb and there is no commitment from council, like you don’t think people are worth investing into”.* They felt this affected morale as:

*“ … we've been slumped as the crappiest suburb in the city. But they're not helping. What are the people going to do against that? They are going - alright you don't want to help us. We are not going to help ourselves*”. (Aboriginal community member)

Other participants described how this lack of investment was perceived by many Aboriginal people in their community as a push to get them out of the area, making people feel *“like no one is investing in their infrastructure because it’s a redevelopment site, and they want black fellas to move out”.* (Aboriginal community member).

#### Abundance and easy access to unhealthy fast food

Participants described how families are surrounded by unhealthy convenience foods in their suburbs; as an Aboriginal Elder described there is “*the fish and chip shop, and the Domino’s pizza - the other pizza up the road. Now that we’ve got McDonalds, a lot of them will walk down to McDonalds”.* As well as being more available, participants felt unhealthy takeaway options were more convenient *“you just ring and order hot chips, that’s it, go in the shop for fresh bread, then go and pick up the chips on the way out and there’s lunch and it feeds them all”,* said an ACCHS staff member. Another participant explained:

“ … *at Dominos you can get pizzas for $6 certain ones and they [children] like those ones. So we can get five or six pizzas and it's cooked and it's about the price of what we would have spent on dinner if we're preparing it all”.* (Aboriginal community member)

Some participants felt that the abundance of fast-food options in their suburb, combined with the view that they are just as cost effective as home cooked meals drives many families to overwhelmingly choose fast food.

#### Limited transport options to access healthy food shops and large supermarkets

Participants described how the lack of affordable, fresh food in their area forces Aboriginal families to travel to the town centre to access the larger supermarkets that have a wider variety of produce and cheaper options. They felt this can be difficult for families without cars or money to run their car, as there was limited public transport in their area and the cost of the bus or a taxi for families on a low income was taking money away from the food budget. Participants explained how limited public transport options in the local area means some people are having to walk home with their groceries, which limits their ability to do a large shopping trip.

Participants also noted that:

“ … *if there’s no buses running at that time, and they can’t get a lift off someone, then they’d have to walk or catch a taxi … on a Sunday when there’s no buses running, that would be difficult”.* (Aboriginal Elder)

Participants explained how some families who have their own transport can go to several different grocery shops to get the cheapest deals, as an Aboriginal community member described *“I’ve got an app on my phone that - I check it before I go shopping”* to look through local catalogues to seek out the best deals on food.

Participants also spoke about how racism plays a role in limiting transport options for families, who struggle to get a taxi to pick them up from their suburb as taxi drivers *“often won’t come to our suburb, unless they know you”* (Aboriginal LHD staff member). Participants felt many families have faced racism on public buses when having to travel with their children and groceries and thought these negative experiences put some people off utilising public transport. Participants described that families who experience mental health concerns had additional challenges in accessing public transport to food shops:

“ … *a lot of people including me with mental health and all that you wouldn't be comfortable getting on a bus. I have that much anxiety about getting lost or climbing on the wrong bus or seeing someone and sitting there either not wanting to talk to them or being scared of someone if you see someone. I know it sounds stupid but that would be an issue for heaps of people”.* (Aboriginal mother)

Participants felt utilising public transport for food shopping was challenging for single parents who often needed to bring along their children which added additional stress and limitations:

“ … *it's hard enough for us to shop without the kids let alone having to go and shop with your kids, take them all on the bus and then have all your shopping. Then once you've got your youngest one out of the pram how do you carry it all? You have to push the trolley then if you're too far away from the shops it's too hard”.* (Aboriginal mother)

#### Lack of stable housing available and cooking facilities

Participants noted that many families do not have access to safe and stable housing, instead having to stay:

*“ … in a car, somebody else’s place, or you couch surf. Under somebody’s house. Like it’s all of those sorts of things are still in play today”.* (Aboriginal LHD staff member)

They felt not having safe and stable housing leaves these community members without access to basic cooking and food storage facilities such as a fridge or a freezer and is a major barrier to being able to prepare healthy foods:

*“ … most of our clients are in Housing. You get the ones that their fridge is broken but don't know that they could get a new fridge pretty fast. Instead they stay without one for months. You get the ones that don't buy food because they might have an infestation of cockroaches and - or their oven's broken, don't have a microwave”.* (ACCHS staff member)

Participants also explained that many families live in older social housing residences which have inadequate storage and bench space in kitchens making it difficult to prepare and store foods from a weekly shop for the family; as an Aboriginal stakeholder explained, *“I’ve got a kitchen maybe half the size of this table”*. They felt the lack of storage meant families were spending more on food as they were doing daily shops, more likely to do more impulse shopping, and were driven towards purchasing easy, take away options.

### Theme 3: limitations of non-Aboriginal food relief services

#### Reliance on food relief and the inflexibility of services

Participants felt many families in their community relied on emergency food relief services and subsidised food boxes:

*“ … they've got Vinnies (St Vincent de Paul Society) and they've got plenty of places where you can go up and get a food order and they won't knock you back. They give you good substantial stuff. It's always meat and veggies and things like that”.* (Aboriginal Elder)

Participants also described the fluctuating nature of food insecurity, where people may lose employment and experience food insecurity for a period of time. As explained by an Aboriginal Elder, *“a lot of people are newly finding themselves in these situations that don’t know how to access [emergency food relief]”*. However, some participants who were employed considered themselves to be the *“working poor”*, making them ineligible for many food relief services. As an Aboriginal mother described, *“I run out of food, and I work. So, I don’t have a healthcare card. I can’t go to St Vincent de Paul and go get a hamper or something like that”.*

Participants expressed that Aboriginal families are generally larger and felt some food relief organizations do not take this into consideration. A participant explained:

*“ … $30 for me and six children … for the single families with two kids it was $20. I don't know how they got a $10 difference with an extra four kids. But that's how they work it out”.* (Aboriginal mother)

Another Aboriginal mother spoke about having to *“wait a week for that appointment”* to get a food voucher, and while some participants acknowledged a week may not seem like a long time, they also noted the challenges of trying to survive for this period with no money or food. Participants also felt the restrictions on food relief services such as time limitations on how regularly you could access food vouchers or hampers made it difficult. As another Aboriginal mother described, “*I don’t want to sound bad or anything like that. I’m appreciative of this … but you cannot get no other help for three months. Three to six months or something like that”.*

#### Stigma and shame linked to accessing non-Aboriginal food relief services

Participants described that Aboriginal people are proud people and felt that many families in their community would not be comfortable receiving free items such as food hampers as *“we don’t want to be looked on as poor or judged”* suggesting that it makes people *“feel shame as they don’t want to be seen as a charity case”,* an Aboriginal Elder explained*.*

Some participants expressed that a number of food relief agencies are either unable or unwilling to try and better understand the Aboriginal community’s needs. Participants described that some families are “*too shame [sic] to say we can’t get enough food because we are overweight”,* consequently they did not feel worthy to go and access food relief services.

Participants explained that food hampers provided by some local charities are often made up of food which is no longer fit for sale. For example, an Aboriginal community member explained how the food is, *“clearance stuff from Woolworths. They go to Woolworths and they get the things that they’re going to throw out. They make them into hampers and then go to disadvantaged communities”.* They described how knowing this makes some families feel disheartened about receiving this food as *“a lot of their stuff is out of date … if you’ve got no food, what’s your option?”*

Participants discussed how some Aboriginal families are reluctant to access food relief services for food hampers or vouchers as:

*“ … people have experienced families and kids being taken away so there is a fear of accessing services as they think you can't provide for your kids so they might get taken way”.* (Aboriginal LHD staff member)

Consequently, participants believed that some parents/caregivers would rather seek assistance from other family and community members than from local agencies, which further added to the financial burden experienced within the community.

### Theme 4: on-going impacts of colonization

#### Cultural identity and food preferences

Participants spoke about how colonization and food rations (such as rice, flour, sugar, tea and fatty meats [53]) received during the mission era (refer to footnote) drastically changed peoples diets, and still impacted on food choices today amongst Aboriginal families in their community [54]. They felt that many foods such as damper (made with white flour), devon (processed luncheon sausage), white bread and hot potato chips are now seen as ‘Aboriginal cultural foods’:

“ … *getting in the mindset of our mob to understand, because we very much see this as cultural food. You know like our devon and potato chips. You know we do think of that like that, but why, and understanding why our diet has changed”.* (Aboriginal LHD staff member)

Participants also explained that their communities were very protective of maintaining these foods and pushing back against modifying them to make them healthier:

“ … *even with the Elders. So in the kitchen we make just your normal damper and just went, oh how about this week we try a half wholemeal and half white flour. It was oh, if looks could kill I would have been dead that day”.* (ACCHS staff member)

*“ … the [ACCHS] had a healthy food policy, and people were so resistant because they thought we were taking away their culture and their practices by saying now you've got to be like white fellas and eat this good way. We were forced like this because of those colonised practices”.* (ACCHS staff member)

Aboriginal community members thought any initiatives that organizations deliver around healthy eating and lifestyle choices should always ensure culture is at the centre underpinning all the activities in their community:

*“ We are like this because of those colonised practices. I think that any programs or things like that, we need to do that education and bringing back the cultural part of it”.* (Aboriginal LHD staff member)

#### Experiences of trauma, racism and disruption to family structures

Participants spoke about the high rates of Aboriginal children being taken from their families, both in the past, during the Stolen Generations period where policies for Australian federal and state government agencies, church missions and welfare bodies to forcibly remove Aboriginal children to be raised in institutions and by white families were in place [55], and currently through removal of children into the ‘out-of-home’ care system with non-relatives [56]. They felt this has led to intergenerational trauma and disrupted family structures:

“ … *the interruption to our parenting practices due to trauma and stress, and the impact, because when you're stressed and trying to feed your family, it is easier to get the two-dollar chips and that”.* (Aboriginal Elder)

Participants also described racism, frequent community deaths, high rates of youth suicide and harassment from police causing constant stress. They felt this stress impacts on motivation to purchase and cook healthy food and leads families to turn to easier meal options as:

*“So if we're struggling, automatically, you're going to go to a fast food shop. So if that's happening maybe two or three times a week within the family, you can guarantee we're going to face obesity, we're going to have diabetes. It's just stress, it's mental health on top of that, yeah. It just affects that whole cycle. That's from the top of our head right down to our toes”.* (ACCHS staff member)

Healthy eating and food affordability were also described as a challenge for parents/caregivers who were suffering with social and emotional wellbeing difficulties themselves or who had children, or multiple family members experiencing inter-generational trauma.

#### Effects of inter-generational poverty on food choices

Participants described how growing up in poverty has made many parents want to ensure their own children have more than they had, which leads them to purchase perceived ‘luxury’ foods they were not able to have as a child, such as soft drinks and takeaway meals:

*“I looked in my cousin's fridge, when I came over once … and I thought oh my God, all these kids have got is water to drink. Those poor little things. I still have that image but now in my head thinking oh my God, my kids were the poor kids. My kids were the ones who were fed the poor nutrition and given that [fast food], because I believed this way. I can remember thinking I should chuck her [my cousin] some more money.* (Aboriginal LHD staff member)

“ … *you see a lot of Aboriginal people, they grew up really, really poor; now that they’ve grown up and they’ve got the money, a lot of them are overweight. Because they can afford that good [luxury] food”.* (Aboriginal LHD staff member)

Participants described how inter-generational poverty has instilled the need to purchase cheap and often unhealthy foods to ration them around the family to survive:

*“When I look back on my life I think about it and I think, was I actually ever hungry? Like we were poor and that, but did I actually go starving and without food. I'm thinking, no, but I lived as if I was a starving child because my father was starving, his grandmother was starving, and food security was in our mindset”.* (Aboriginal LHD staff member)

#### Generational loss of healthy food knowledge and preparation skills

Aboriginal Elders spoke about how they have observed a generational gap in knowledge in the younger generation in their community about how to cook cheap, quick and healthy meals, which they felt was owing largely to children being removed and experiencing associated trauma:

“ … *you know that generational slippage when there is trauma or interrupted parenting practices, kids removed, coming back in, this is stuff [healthy meal preparation] we learnt but now how are we passing that and teaching that on?”.* (Aboriginal Elder)

*“ … it’s the young mums; you need to target the young mums, because all of a sudden, they’ve got a baby. It’s all well and good when they’ve giving them the formula and then the baby food, but then: okay, it’s time to start eating real food, what do you feed the baby?”* (Aboriginal Elder)

Conversely, some participants felt that some parents/caregivers lack knowledge about healthy foods and how to cook them, as an ACCHS staff member explained, *“I know kids that don’t even eat fruit and veg. It’s sad that the parents aren’t instilling that into their kids from a young age”.* Participants also noted that parents/caregivers may not have skills in how to budget, prepare for meals or to write up a weekly shopping list. This gap in budgeting and meal preparation skills was thought to cause many families in the community to spend more money as they are shopping daily:

“ … *if families are taught meal prepping, young families, that you can have these meals in your freezer, whether you have two the next week or something, or it’s lunch the next day or dinner the next night. It’s something that young people need to be taught”. (*Aboriginal Elder)

Overall, participants thought that group education led by the Aboriginal community would help build skills in young families to budget, prepare for and cook healthy meals and help to ensure these skills would be passed on to future generations:

*“Well, I think the community have to get off their backside too and think about what can we do to make a better community … We need to think about things that are not too difficult for people to take on together. Even if it's just starting a little garden and then it grows. You remember that song, from little things big things grow”.* (Aboriginal Elder)

*“I think it [programs] would be better in groups and then they lift each other up, rather than just going by yourself, it's a lonely world. I think we need to do that by community groups”.* (Aboriginal Elder)

### Theme 5: maintaining family, cultural and community commitments and responsibilities

#### Sharing resources amongst family and community

Participants discussed the unique kinship networks with extended family that exist in Aboriginal cultures. They explained this means families are often providing food and resources not just to people within their household but amongst their wider kinship and community networks, which is not typical amongst non-Aboriginal people:

“ … *sometimes there'd be about 17 plates I'll dish out because I'm feeding for me and my family, my sisters, my nephews, my niece, other kids that come there and stay with us”*. (Aboriginal community member)

*“I've had heaps of people come and live with me because they've got nowhere to live, and they're not on Centrelink or anything like that so they can't help contribute. But you've still got to feed that extra mouth sort of thing. You can't turn them away. So you and your family are struggling as well as the other person really. So I think that's the major thing, is taking on board more people than you can afford to feed sort of thing”.* (ACCHS staff member)

Sharing of food and resources was frequently described as a positive way to manage food insecurity within the community:

*“ … because of the way Aboriginal families support each other - if one household has run out of food, then they can go and see another family member who can assist them, whether they give them food, or they assist them to get access to food”*. (Aboriginal LHD staff member)

Participants also explained how the unique kinship networks and connection with the wider Aboriginal community meant that Sorry business (funerals and cultural practices and protocols associated with death) happens quite regularly. Consequently, families have lots of mob (family) staying with them, which is often unplanned, making it hard to budget and prepare for as *“you don’t know how many people are going to turn up to your door for a feed”,* an Aboriginal mother explained. In some instances, mob might stay for only a few nights or sometimes for months, which increases both parties’ financial stress. Participants felt that government payments and food relief agency funding failto take this into consideration, leaving many families further disadvantaged. However, a participant explained:

*" … the AMS [Aboriginal Medical Service] will then try to assist through its various programs. If it is Sorry Business - and we know a lot of people are going to that house, then we will assist by dropping off tea, coffee, stuff like this, some items that keep them going whilst they’ve got a houseful”.* (ACCHS staff member)

*“ … well you know for an example, this morning, a community member just passed on. That means that the whole community will come together that are linked with this family, because of the stresses that the family are under now, the wider the community comes together to be able to provide those meals for those families”.* (ACCHS staff member)

### Fulfilling other family responsibilities

Participants felt families are increasingly busy with children’s after school activities which left less time for cooking meals at home. They described how the time pressures families face are driving them towards looking for quick and easy options for meals:

*“So families with young kids that do sports and have extra curricular activities, a lot. Because by the time school's done and you're running to sports and what not, it's about 7:30, eight o'clock and it's easy to grab your big four. Macca's, KFC, Red Rooster and Hungry Jack's”.* (ACCHS staff member and mother)

Participants explained that many Aboriginal grandparents, aunties, uncles and other family members are caring for additional children, particularly where social and emotional well-being issues were affecting the family. They felt this put financial strain on those family members, which was hindering their ability to afford enough food:

“ … *a lot of the time grandparents are doing it, and there are so many kids in the household that it's so hard to look after everybody and feed everybody, and with the depression and anxiety on top of that, and sometimes drug addictions and things like that, they're just [keeping everyone alive*]”. (Aboriginal Education staff member)

### Recommendations for local action

Participant suggested a number of potential solutions to help ease food insecurity in the two communities, including the introduction of school breakfast programs, cooking and budgeting programs in the ACCHSs and schools, community vegetable gardens, increased transport options for the community and establishment of subsidised food programs that cater to the communities’ needs. Aboriginal community members also felt strongly that Aboriginal culture and values should be at the heart of new food security initiatives, stating that it would positively impact on community participation, mental health, and overall wellbeing.

## Discussion

This study gathered community and stakeholders perspectives on the drivers of food insecurity and their recommendations in terms of potential strategies that could alleviate food insecurity experienced by Aboriginal communities located in an urban and a large regional area in NSW. Study participants reported that food insecurity was a huge problem experienced by the families in their communities on a regular basis. There were several key factors identified in our study, which appear unique to food insecurity as experienced by Aboriginal communities in non-remote settings. These were factors linked to colonization, cultural identity, racism and stigma when accessing mainstream organizations for food relief, as well as those linked to the food system such as a lack of adequate infrastructure in the local food environment in the areas where Aboriginal communities reside. The reported positive role of kin and community in sharing money and food with families struggling with food insecurity in this study has been highlighted in other work with Aboriginal populations [57, 58].

Other drivers of food insecurity described by the participants were consistent with food security literature in Australia and internationally. These included, high rates of unemployment and low household income [58], lack of transport options to purchase food [58–61] and distance to large supermarkets selling a greater variety of less expensive produce [59, 62–64]. Time pressures combined with a lack of nutrition knowledge [65], cooking and budgeting skills drove families towards easy meal options [66–68]. As found in other studies with Aboriginal people and Indigenous peoples globally, unstable and inadequate housing and lack of home utilities, were issues impacting on families' ability to be food secure [69–72]. The findings reported in this study were consistent between the urban and regional study sites.

Financial barriers, commonly reported among Aboriginal communities, were described as a major factor impacting on participants' food security [16, 22, 58, 69]. The perceived high cost of healthy food reported in this study has been observed in other studies in Aboriginal communities [58, 69, 73–75]; however, most of these studies have been conducted in remote areas, with relatively little price data available from urban areas [16, 75, 76]. A study conducted in Western Sydney that investigated the affordability of a food basket (incorporating principles of health and sustainability) showed that the most disadvantaged groups in the region, both at the neighbourhood and household level, experienced the greatest inequality in affordability of the healthy and sustainable diet [17]. Another recent study in regional NSW highlighted that price alone is not the major factor but that income combined with other factors impacts on the affordability of food [77]. The study compared the average cost of the Victorian Healthy Food Basket [78] with the same food basket in regional NSW, and found that 34% of a four person family’s government income support payments went towards purchasing the basket indicating considerable food stress in these households [77]. Evidence from other studies show that households on low incomes spend a greater proportion on food than those on higher incomes [19, 75] and those living in the most socio-economically disadvantaged areas are the most impacted [16, 79]. Data from remote Aboriginal communities indicate higher levels of food unaffordability [79].

Study participants highlighted the limited availability of fresh, healthy food and the greater concentration of fast-food outlets in their suburbs as well as difficulties with public transport which made it harder for them access larger supermarkets that sell a variety of cheaper and healthier food. Neighbourhoods play a key role in health [80] and it must be noted that efforts made by Aboriginal families to eat healthy may be compromised by an unhealthy food environment. Data from this study supports evidence from other research that shows that food environments in disadvantaged areas are less healthy than those in affluent areas [10, 69, 77]. A recent study conducted in Sydney, Australia, identified so-called 'food deserts' and 'food swamps' in the city’s economically disadvantaged areas where healthy food options were scant or non-existent while unhealthy food was easy to purchase when compared to the more affluent suburbs [23]. It also reported that the rates of type 2 diabetes were up to 3 times greater in the disadvantaged suburbs, confirming that greater exposure to unhealthy food can negatively influence dietary behaviour, confirming the role of socio-economic status and racial disparities in chronic disease risk as has also been noted from studies conducted in the United States [81]. These findings highlight the importance of the need for public policies related to the food environment which prioritize areas with poor availability and access to healthy food to overcome inequality.

Other research supports what participants reported regarding older social housing dwellings, with limited storage and cooking facilities hampering their ability to cook healthily at home, particularly for large families [69, 71]. Studies have shown that Aboriginal people are four times more likely to live in social housing [82–84], three times more likely to live in run down houses in need of repairs [85] and 14 times more likely to have experienced homelessness than non-Aboriginal people in Australia [86]. Research has shown that social housing in Australia has generally been mass produced, poorly designed and often located in the outskirts of town centres. As found in other studies with Aboriginal people and other Indigenous peoples globally, stable and adequate housing was an issue impacting on a family’s ability to be food secure [70–72].

Colonization has impacted on all aspects of Aboriginal peoples’ lives, through marginalisation, economic exclusion, and barriers to educational attainment, which has led to families experiencing lower incomes, living in social housing and in areas with poorer infrastructure [87–89]. This has resulted in higher rates of incarceration, homelessness, youth suicides, and experiences of racism and inter-generational trauma from the forced removal of children [89, 90]. The on-going impacts of colonization play a large part in food insecurity experienced by Aboriginal communities, with drastic changes in their local food systems. Aboriginal people were forced off Country (traditional lands) and made to live on missions and reserves (see footnote) where they were not permitted to maintain a traditional diet. Mission and reserve life forced Aboriginal people to consume an energy dense, ‘Western’ diet, provided by food rations such as white flour, rice, poor quality meat, sugar, tea, alcohol and tobacco [53, 57]. In our study, this disruption was viewed as still currently impacting food choices made by many Aboriginal families today due to a change of taste preferences from traditional foods to ‘Western’ diets, in addition to the loss of traditional food sources and knowledge on how to locate and prepare them. Future programs aimed at healthy eating need to take into consideration these impacts of colonization on Aboriginal peoples' diet and food choices. Aboriginal families also described disruption to family structures as a result of the Stolen Generations [56] and children currently being placed in out of home care to have greatly impacted on important nutrition knowledge and cooking skills being passed on to children. Nutrition research conducted with young people leaving the out-of-home care system showed that most were consuming a diet predominately of foods with low nutritional value [91]. Participants reflected on how the challenges that past generations experienced are impacting purchasing patterns in their communities. For instance, as a result of many generations within a family living in poverty and having to purchase cheap food to ration around family members, a behavioural pattern has been instilled; in families who now may no longer be experiencing poverty that behaviour instinctively remains. On the other hand, for some parents/caregivers this has led them to wanting to provide their children with perceived luxury items such as takeaway, soft drinks and packaged lunchbox items, items they themselves were not able to have as children.

Charities and food relief programs can play an important role in aiding families on low incomes who are experiencing food insecurity [77, 92]; however, feelings of shame and fear of judgement can prevent some families from accessing these services [66, 67, 93]. We would argue that these feelings are even stronger barriers amongst Aboriginal communities [94] as many people have experienced and continue to experience family members being removed from the family at alarming rates [56]. Understandably, fear was particularly noted when accessing non-Aboriginal food relief organizations, which has been described in literature as a major barrier for Aboriginal people in accessing mainstream health services more generally [95–97] and also amongst Indigenous peoples globally [98]. A lack of compassion and understanding for the trauma and on-going racism Aboriginal people face when accessing mainstream services was also described, which is consistent with other studies amongst Indigenous populations [98, 99]. One potential solution would be for mainstream organisations to undertake a critical reflection of their practices to gain an understanding of how racism (both past and present) impacts Aboriginal peoples’ interactions with their own programs [100].

A number of coping strategies such as rationing, skipping meals and buying cheap unhealthy food to fill kids up, are highlighted in this study and have also been found in other studies in both Aboriginal populations and other populations internationally [69, 93, 101]. The sharing of food and resources amongst family and the wider Aboriginal community was also described as a way families deal with an inadequate income and was viewed positively as an important cultural practice. Sharing as a cultural practice is an important part of continuing social connectedness amongst family and the wider Aboriginal community and has been discussed in other literature [58, 102, 103]. It was mentioned as a key strategy used to deal with food insecurity in our study, which reflects findings in other studies on Aboriginal people [58, 68].

Our study brings to light, for the first time, a number of unique factors that affect Aboriginal peoples’ food security and food choices, several of which are colonial legacies that continue to impact upon Aboriginal peoples today. Therefore, when developing food security programs Aboriginal ways of being, knowing and doing need to be at the core to ensure the program is relevant and addresses these factors [100]. By co-creating programs with Aboriginal communities this ensures they encompass important factors often not considered, such as, culture, identity, and family and community relationships and connection.

### Strengths and limitations

Using decolonising methodology allowed the study to privilege the voices of Aboriginal community members and enabled the research to explore experiences and draw out deeper issues impacting food security that a Western approach to research may not have been able to.

Participants were recruited through ACCHSs and not food relief organizations, which allowed for a wider range of perspectives and not just of those accessing food assistance programs. Each interview had an Aboriginal researcher with at least 10 years’ experience working in ACCHSs, which shaped the meaning of the data collected and guided the approach to analysis. This played a positive role in the communication of the interview questions and in establishing trusting relationships in which people felt comfortable to share personal experiences. Having Aboriginal people guide and lead the research across all aspects enabled the data to be collected, interpreted and written from a standpoint not available to non-Aboriginal researchers, and therefore, led to a more accurate story of food insecurity.

The following limitations should be considered in relation to the findings from this study. Firstly, the data were collected with two Aboriginal communities, one in an urban area and another in a large regional area of NSW. Aboriginal communities throughout Australia are diverse, so the transferability of the findings to other populations and setting is uncertain. The findings from this study may resonate with other Aboriginal communities in urban and large regional areas, as there were similarities with other study findings in similar settings. Secondly, although this study approached several stakeholders from the food retail and supply industry, we only had one agree to participate in the project.

## Conclusions

This study found that Aboriginal families in urban and regional areas in Australia are experiencing food insecurity on a regular basis, which is impacted by a range of socio-economic, environmental, systemic and cultural factors, as reported by the study participants. The study identified several contributing factors including poverty and unemployment, inequities in the availability and accessibility of affordable healthy food, stigma and racism, and generational loss of healthy food knowledge.

Our findings highlight the importance of Aboriginal culture, identity, and family and community relationships. The sharing of food and resources amongst family and the wider Aboriginal community was the main way in which people dealt with food insecurity and was overwhelmingly viewed as a positive cultural factor. The sharing of food and resources was of a reciprocal nature where families would help each other out and that the favour would be returned when needed. Food relief charities were another way many Aboriginal families accessed support in dealing with food insecurity on a short-term basis.

Further work is needed to explore several important areas impacting on Aboriginal food insecurity, such as the local food environment and food system as well as culture, traditional foods, and the on-going impacts of colonization that impact food choices and consumption, and Aboriginal food sovereignty. There is a need for partnerships to be strengthened between Aboriginal organizations, local government, food suppliers and the health sector and co-ordinated efforts to drive the co-design of culturally appropriate recommended programs and policies to improve food security and health outcomes for Aboriginal people.

## Supplementary Information

**Additional file 1.**

## Data Availability

The qualitative data set used during the current study is available from the corresponding author on request.

## Abbreviations

ACCHS: Aboriginal Community Controlled Health Service
NGO: Non-Governmental Organization
Vinnies: St Vincent de Paul Society, a Catholic aid agency
LHD: Local Health District
