# Supplementary material for: Murradambirra Dhangaang (make food secure): Aboriginal community and stakeholder perspectives on food insecurity in urban and regional Australia
Source: BMC Public Health. 2022 May 28;22:1066. doi: 10.1186/s12889-022-13202-z (PMC9146813; doi:10.1186/s12889-022-13202-z)
Supplement: Supplementary file 1 — Additional file 1. [file 12889_2022_13202_MOESM1_ESM.docx]

**Supplementary file 1: A systems perspective on the prevention of chronic disease for urban Aboriginal communities: Improving food security**

*A. Interview Guide for parents and carers of Aboriginal children*

**Participant demographics**

1. Name: _________________________________________
2. ACCHS: ________________
3. Age in years ________________
4. Sex: Male Female
5. Aboriginal and/or Torres Strait Islander Yes No

6. What best describes your current relationship status? (Tick one)

Single, never married

Living with partner

Married

Separated

Divorced

Widowed

7. What is the highest level of education that you have completed (tick all that apply)

No formal schooling

Primary school

High school

Further training/ apprenticeship

Certificate

Diploma

Bachelor’s Degree

Postgraduate degree/ certificate

8. Employment

Full time

Part time ______ (hours/week)

Not employed

Home duties

9. Do you receive any of the following benefits? (Tick all that apply)

Disability support

Sickness benefits or allowance

Family allowance

Repatriation pension or service pension

Carer’s allowance (for caring with a child with a disability or major health problem)

Unemployment benefits

Parenting payment

Age pension

10. How would you say you are managing financially at the moment?

Living very comfortably

Living quite comfortably

Getting by

Finding it quite difficult

Finding it very difficult

11. Is your current home:

Owned outright by yourself

Owned with a mortgage by yourself

Rented privately

Rented from Department of Housing/ Aboriginal Housing Office/ Community Housing provider

12. What best describes your current house?

House

Unit

13. How many people currently live in your home? Please specify number:

Adults

Children

**Introduction to study**

- This project is about ***understanding*** the problem that Aboriginal people may face, which is running out of food sometimes
- Our aim is to hear about your views and experiences in this area so that the information we gather may be of benefit to you and your community
- We would therefore appreciate your honest answers to a few questions we have for you
- There are no right or wrong answers and you won’t be judged by your responses
- We want to ask you about your thoughts on healthy food: particularly on *healthy food* being *available, easy to get, affordable and acceptable* in your community to help us figure out ways we can work with the local AMS, local council, NGOs, schools, and other organisations in your area to improve this.
- We will ask you about how available healthy food is in supermarkets, school canteens, restaurants, fast food outlets, take away shops and eateries in your area. We want to find out how you get to these places, how often you go, where they are located, and why you go there.
- The information that we collect from this project will help us identify areas where there are gaps in healthy food being available, easy to get, affordable and acceptable for you and your community.
- All the information you provide is confidential – None of the information will be passed to any organisation or anyone and your name or identity will not be used in any of the reports, publications or materials arising from this work.

**Questions:**

1. In the past 12 months, was there any time when you and your household ran out of food?
2. In the past 12 months, was there any time when you and your household were worried about running out of food?

*(Skip questions 3-6 if answer to Question 1 and 2 was No)*:

1. When this happened, did you and your household go without food? How long for?
2. How do you and your household cope with running out of food? What do you do? **Prompt:** skip meals, family/friend networks, food banks and vouchers, charities.
3. Can you please explain some of the reasons why you and your household ran out of food or have been worried about running out of food? **Prompt**: food not available, difficulty getting food, cost?
4. Are there certain times of the week, fortnight, month or year when this occurs? Or does something else happen regularly that causes you to run out of food or to worry about running out of food? (**Prompt**: medical emergencies, large bills, helping family members with their needs, changes in job status)
5. Now let’s talk about healthy food: How would you describe what healthy food is? Could you name some healthy foods? **Prompt***:* if respondent talks only about fruit and vegetables, probe for other foods such as whole grain food, beans, lean meats (specify type of meat eg: mince, sausage, steak etc), eggs, chicken, fish, milk, yoghurt
6. Is healthy food easily available in your local area (markets, retailers, food outlets)? If not, what do you see are the problems? If yes, can you tell us about these places and what is available there?
7. Where do you usually go to buy groceries for your household? (non-fresh produce)

(**Prompt:** Supermarket, grocery store, general store, corner store, petrol station, farmers market, online)

1. Where do you usually shop for fresh food for you and your household? (vegetables and fruit, meats, fish and chicken) (**Prompt:** Supermarket, grocery store, general store, convenience store, petrol station, farmers market, own garden, community garden, online, farm animals)
2. Are there any foods that are not available in your local area which you would like to have?
3. Are you able to get enough of the healthy food you and your household need? **Prompt:** what prevents you from getting enough; cost etc
4. Are there any things that make it difficult for you to get to markets or shops or food outlets that supply healthy food? **Prompt**: shops are far away; don’t have a car
5. Do you face other difficulties at home that make it harder for you and your household to eat healthy food? **Prompt:** food storage, appliances and cooking skills
6. What makes you choose the food you normally eat? Overall, how healthy would you say the food you and your household eat at home is? **Prompt**: time, preparation, convenience, cost, nutritional value/health, family/cultural preference, fussy eaters and advertising?
7. For the shops/eateries that you use most often, how far away are they? How do you usually get to these shops/eateries? Is there reliable, affordable public transport to and from the food stores that you want to shop at? **Prompt:** Cafes, restaurants, take away etc
8. Overall, what do you think about the cost of food in your area? I’m interested in your views on the affordability of healthy food items like fruits, vegetables, bread, milk, whole grain foods, eggs, meat, chicken etc in your local area.
9. How often do you or your household get take away food or eat at fast food outlets? What are the main places you often go? What is the main reason you get these foods?
10. What do you think can be done to make it easier for Aboriginal people in your community to be able to buy and prepare enough (healthy) food? **Probe for:** what the following groups can do: Community, council, schools, individuals
11. Can you please tell us what types of programs would support your community to get enough of the healthy food you and your community need?

**Many thanks for your time**

**A systems perspective on the prevention of chronic disease for urban Aboriginal communities: Improving food security**

*B. Draft Interview Guide for Aboriginal Community Controlled Health Service staff*

**Participant demographics**

1. Name: ________________________________
2. ACCHS: _____________________
3. Role in ACCHS: _____________________
4. Years at ACCHS: ________________

1. Age in years ________________

1. Sex: Male Female

7. What is the highest level of education that you have completed (tick one or more)

No formal schooling

Primary school

High school

Further training/ apprenticeship

Certificate

Bachelor’s Degree

Postgraduate degree/ certificate

8. Employment

Full time

Part time ______ (hours/week)

**Introduction to study**

- This study is about ***understanding*** a problem that Aboriginal people face – that of running out of food sometimes - and the various factors that might contribute to it.
- We also want to talk about the different aspects of ‘***food security***’ - which includes ***healthy food being available, easy to get, affordable and acceptable*** in order for people to live healthy and active. We would like to explore these aspects of food security in your community here.
- We will ask you about how available healthy food is in supermarkets, convenience stores, farmer’s markets, school cafeterias, restaurants, fast food outlets, take away shops and the like in your area.
- We will ask you questions about location of food stores or eating places, how Aboriginal people generally get there.
- We will also ask you questions about what you understand by culturally acceptable healthy food.
- The information that we collect from this study will help us identify areas where there are gaps in healthy food being available, easy to get, affordable and acceptable for your community.
- All the information you provide is confidential – None of the information will be passed to any organisation or anyone and your name or identity will not be used in any of the outputs from this work.

1. Do you think that Aboriginal and Torres Strait Islander people sometimes run out of food or are worried they would run out of food?

(a) Is this happening in the homes of your family and friends?

(b) Is this happening in the wider Aboriginal and Torres Strait Islander community in Wagga Wagga?

*(Skip questions 2-6 if answer to Question 1 was No)*:

1. When this happens, do members of Aboriginal households go without food?
2. How do Aboriginal households cope with running out of food? What do they do?
3. Can you please explain some of the reasons why Aboriginal households run out of food or have been worried about running out of food? **Probe for** – food not available, difficulty getting food, cost?
4. Are there times when Aboriginal households run out of food and cannot afford to buy more food?
5. Does running out of food follow any pattern in your communities? Are there specific times of the week or fortnight or month or year when this occurs? Or does something else happen regularly that causes Aboriginal households to run out of food or to worry about running out of food? (**Probe for**: medical emergencies, large bills, helping family members with their needs, changes in job status)
6. Now let’s talk about healthy food: What do you understand by healthy food? **Probe for***:* if respondent talks only about fruit and vegetables, probe for other foods such as whole grain food, beans, lean meats, eggs, chicken, fish, milk, yoghurt
7. Is healthy food easily available in your local area (markets, retailers, food outlets)? If not, what do you see are the problems?
8. Where do Aboriginal families usually go to buy your grocery for their household?

(**Probe for:** Supermarket, grocery store, general store, convenience store, petrol station, farmers market, internet)

1. Where do Aboriginal families usually shop for fresh food for their household? (vegetables and fruit, meats, fish and chicken) (**Probe for:** Supermarket, grocery store, general store, convenience store, petrol station, farmers market, own garden, community garden, internet, farm animals)
2. Do Aboriginal families get enough of the healthy their households need?
3. Are there any foods that are not available in your local area which Aboriginal families would like to have?
4. Are there any things that make it difficult for Aboriginal families to get to markets or stores or food outlets that supply healthy food? **Probe for**: shops are far away; don’t have a car
5. For the stores that Aboriginal families use most often, how far away are they? How do they usually get to these stores? Is there reliable public transportation to and from the food stores that they want to shop at?
6. Overall, what do you think about the cost of food in your area? I’m interested in your views on the affordability of healthy food items like fruits, vegetables, bread, milk, whole grain foods, eggs, meat, chicken etc in your local area.
7. How often do Aboriginal families get take away food or eat at fast food outlets such as McDonald’s, KFC, Hungry Jacks, Pizza Hut, Domino’s? What is the main reason they get these foods?
8. What makes Aboriginal families choose the food they normally consume? **Probe for**: time, preparation, convenience, cost, nutritional value/health, family/cultural preference, and advertising?
9. What do you think can be done to make it easier for people in your area to get and use enough (healthy) food?

Probe for what the following groups can do: Community, council, schools, individuals

1. Can you please tell us what types of interventions that would enable your community to get enough of the healthy food they need?

**Many thanks for your time**

**A systems perspective on the prevention of chronic disease for urban Aboriginal communities: Improving food security**

*C. Interview Guide for stakeholders in the food supply process*

**(Local Council, Government, NGOs, Food growers and retail outlets)**

**Participant demographics**

1. Name: __________________________________________________________
2. Organisation: ____________________________________________________________
3. Role in Organisation:__________________________________________________________
4. Age in years ________________
5. Sex: Male Female

6.What is the highest level of education that you have completed (circle one)

□ No formal schooling

□ Elementary school

□ Middle school grade left school _____________

□ High school

□ Some college

□ Bachelor’s Degree

□ Master’s degree or higher

**Introduction to study**

- This project is about ***understanding*** a problem that Aboriginal people and other vulnerable communities in your local area may face - *that of running out of food sometimes*
- Our aim is to hear about your views and experiences on this subject so that the information we gather may be of benefit to people your local community.
- There are no right or wrong answers, so please feel free to express your thoughts
- We want to ask you specifically about your thoughts on healthy food: particularly on *healthy food* being *available, easy to get, affordable and acceptable* in your community. This will help us figure out ways we can work with the local council, Government, NGOs, charity organisations, schools, local Aboriginal Medical Services, and other organisations in your area to improve this.
- We will ask you about how available healthy food is in supermarkets, shops, school canteens, restaurants, fast food outlets, take away shops and eateries in your area. We want to find out how Aboriginal people get to these places and how often they go, where the shops are located, and why the Aboriginal people choose the shops they go to.
- The information that we collect from this project will help us identify areas where there are gaps in healthy food being available, easy to get, affordable and acceptable for vulnerable people living in the local area. E.g Aboriginal families.
- All the information you provide is confidential – None of the information will be passed to any organisation or anyone and your name or identity will not be used in any of the reports, publications or materials arising from this work.

**Questions:**

1. Are you aware that Aboriginal households (and other disadvantaged groups?) in your local area sometimes run out of food or are worried they would run out of food?

*(Skip questions 2-5 if answer to Question 1 was No)*:

1. When this happens, would you know what these households in your local area do? Do they go without food? If yes, for how long?
2. Do you have any thoughts on how these families generally cope with running out of food? What do they do? **Prompt:** services they access for help, borrow money or food from family/friends?
3. Would you know what might be some of the reasons why these Aboriginal families run out of food or have been worried about running out of food? **Prompt**: food not available, difficulty getting food, cost?
4. Are there certain times of the week, fortnight, month or year when this might occur? Or does something else happen regularly that causes some of the families in your local area t run out of food or to worry about running out of food? Prompt: medical emergencies, large bills, helping out family, changes in job status.
5. Now let's talk about healthy food: How would you describe what healthy food is? Could you name some healthy foods? Prompt: if respondent talks only about fruit and vegetables, probe for other foods such as whole grain food, beans, lean meats (specify type of meat eg: mince, sausage, steak etc), eggs, chicken, fish, milk, yoghurt.
6. Is healthy food easily available in your local area (markets, retailers, food outlets)? If not, what do you see are the problems? If yes, can you tell us about these places (including yours if it is food market or shop) and what is available?

S*pecific questions for food retailers and Charity organisations (and potentially those in local government and council):*

1. Would you know where Aboriginal families in your community usually go to buy groceries for their household? (Non-fresh produce). **Prompt**: Supermarket, grocery store, general store, corner store, petrol station, farmers market, online.
2. Would you know where Aboriginal families in your community usually shop for fresh food for their household? (vegetables and fruit, meats, fish and chicken) (**prompt:** Supermarket, grocery store, general store, corner store, petrol station, farmers market, own garden, community garden, online, farm animals)
3. Can you tell us about organisations that supply alternative, cheaper food in the local area? Who organizes them and what do they sell/supply?
4. Do Aboriginal families buy food at these stores regularly?
5. Do Aboriginal families get ‘enough’ of the healthy foods their households need? Prompt: what prevents them from getting enough eg: cost
6. Are there any things that make it difficult for families in your community to get to markets, shops or food outlets that supply healthy food? Prompt: shops are far away; don't have a car
7. For the shops that Aboriginal families use most often, how far away are they from where these communities live? How do they usually get to these stores? Is there reliable, affordable public transport to and from the food stores that they want to shop at?
8. What do you think can be done to make it easier for people in your area to get and use enough (healthy) food? Prompt: what can the following groups do: Community, council, schools, individuals
9. Are there any farmland preservation efforts or community garden initiatives in your area?
10. Do you think there is a good balance between eateries that sell healthy food versus unhealthy food in your area?
11. Overall, what do you think about the cost of food in your area, keeping in mind Aboriginal families that may be doing it tough? I'm interested in your views on the affordability of healthy food items like fruits, vegetables, bread, milk, whole grain foods, eggs, meat, chicken etc in your local area.
12. How often do Aboriginal and other disadvantaged families in your community get take away food or eat at fast food outlets? What are the main places families purchase this from? What is the main reason they get these foods?
13. Are there any policies that limit the number of food outlets that sell unhealthy food in your area?
14. Do you have any recommendations on what else could be done to improve the Aboriginal community’s problems with food insecurity?
15. Are there local funding sources for community food security-related activities?
16. What do you see as the specific role of your organisation and other stakeholders in improving availability, accessibility and affordability of healthy food among Aboriginal people and other vulnerable groups in your area?
17. What do you see as the most useful ways to create synergies among different stakeholders on improving availability, accessibility and affordability of healthy food particularly for Aboriginal families and other disadvantaged groups in your area?

**Many thanks for your time**
